# Topological and functional analysis of nonalcoholic steatohepatitis through protein interaction mapping

**Published:** 2016-12

**Authors:** Hamid Asadzadeh-Aghdaee, Vahid Mansouri, Ali Asghar Peyvandi, Fathollah Moztarzadeh, Farshad Okhovatian, Farhad Lahmi, Reza Vafaee, Mohammad Reza Zali

**Affiliations:** 1 *Basic and Molecular Epidemiology of Gastrointestinal Disorders Research Center, Research institute for Gastroenterologyand Liver Diseases, Shahid Beheshti University of Medical Sciences, Tehran, Iran*; 2 *Proteomics Research Center, Shahid Beheshti University of Medical Sciences, Tehran, Iran*; 3 *Hearing Disorders Research Center, Shahid Beheshti University of Medical Sciences, Tehran, Iran*; 4 *Department of Biomedical Engineering, Amirkabir University of Technology, Tehran, Iran*; 5 *Physiotherapy Research Centre, School of Rehabilitation, Shahid Beheshti University of Medical Sciences, Tehran, Iran*; 6 *Behbood Gastroenterology and Liver Diseases Research Center, Shahid Beheshti University of Medical Sciences, Tehran, Iran *; 7 *Gastroenterology and Liver Diseases Research Center, Research Institute for Gastroenterology and Liver Diseases, Shahid Beheshti University of Medical Sciences, Tehran, Iran*

**Keywords:** Fatty liver disease, Protein-protein interaction Network, Cytoscape

## Abstract

**Aim:**

The corresponding proteins are important for network mapping since the interaction analysis can provide a new interpretation about disease underlying mechanisms as the aim of this study.

**Backgroud:**

Nonalcoholic steatohepatitis (NASH) is one of the main causes of liver disease in the world. It has been known with many susceptible proteins that play essential role in its pathogenesis.

**Methods:**

In this paper, protein-protein interaction (PPI) network analysis of fatty liver disease retrieved from STRING db by the application of Cytoscape Software. ClueGO analyzed the associated pathways for the selected top proteins.

**Results:**

INS, PPARA, LEP, SREBF1, and ALB are the introduced biomarker panel for fatty liver disease.

**Conclusion:**

It seems that pathways related to insulin have a prominent role in fatty liver disease. Therefore, investigation in this case is required to confirm the possible linkage of introduced panel and involvement of insulin pathway in the disease.

## Introduction

Nonalcoholicfattyliverdisease(NAFLD)couldbeclassified as relatively benign simple steatosis up to progressive nonalcoholic steatohepatitis (NASH) as a common chronic liver disease (1). The increasing incidence of the disease is 178% in adolescent populations (2). NASH is one of the liver diseases that some patients may not aware about its signs and symptoms. It is characterized by fat in liver accompanied by inflammation and damage (3). NASH is similar to alcoholic liver disease, however the patients may drink little alcohol or may not (4). The disease may finally lead to cirrhosis and sever damages in patients (5).The condition that people have fat in their liver without inflammation and other clinical symptoms is known as fatty liver(1) however, NASH has been difficult to understand and treat for both scientists and clinicians (6). NAFLD is diagnosed by liver scan to show fat in liver and other tests (7). Biopsy is required for differentiation between simple fatty liver and NASH (8) as an invasive method therefore, noninvasive serum biomarkers for evaluation of liver disease and fibrosis were presented (9). Occurrence of NAFLD in people at 40-50 year old were reported(10)and this age is a risk factor for heart disease accompanied by obesity and type 2 diabetes(11). Hooper et al presented mutations associated with increasing lipid synthesis and uptake or decrease in hydrolysis or export involved in NAFLD (12). The heritability of NAFLD has been demonstrated to be approximately 39% comparing the presence of fatty liver in siblings and parents of patients (13). There are differences in NASH prevalence between male and female (14) and different races as increased in Hispanics compare to other races (15). Molecular investigation for NASH disease showed that there are many contributing genes and proteins in NASH pathogenesis (16, 17).

Some Genes and proteins involved in NAFLD was summarized:

1. *Gene APOc3 *with protein “Apolipoprotein C3” is a surface component of VLDL and inhibits LPL(18).
2. *Gene ATGL *function is to catalyzes the initial step in triglyceride hydrolysis and “Adipose triglyceride lipase “ protein is involved with ATGL(19)
3. *Gene CGI-58 *is an activator of triglyceride hydroxylases. It works along with “Comparative Gene Identification-58” protein.
4. *Gene GCKR *down regulates the glucokinase J with “Glucokinase regulatory Protein “participation (20).

*5-Gene LXR *with “Liver X Receptor” protein is a transcription factor for numerous target genes involved in glucose and lipid metabolism (21).

An interaction view of how these proteins relate to each other can support further associations for some of the specific ones. These specific elements are known as central proteins that are analyzed through network centrality examination. The term used for these key proteins is hub-bottlenecks. It is established that malfunction of each one of these key proteins can be the main reason for any abnormal conditions such as disease phenotypes. Consequently, PPI network construction as the aim of this study could be helpful to determine these fundamental agents in NASH for the better understanding of the disease.

## Material and Methods

The network construction for fatty liver disease was through Cytoscape Software (22) and by the application String database (db). String is a database of known and predicted protein interactions. The interactions are retrieved from four sources including genomic context, high-through put experiments, (conserved) co expression, and previous knowledge. String db has three options for providing information, including protein query, PubMed query, and disease query. Here, disease query was chosen for retrieving proteins related to Nash Disease. The proteins that were obtained from disease query have associated disease scores. The disease score shows that how much the protein is linked to the disease based on different sources such as experimental and text mining. A number of 100 proteins (nodes) with combined confidence score cutoff of 0.4 were considered for this query. Following network construction, the corresponding network topology parameters were determined by the use of Network Analyzer, which is well integrated in Cytoscape. The two important parameters examined in this study is degree and betweenness centrality (BC). The proteins with high degree are known as hubs while proteins with high betweenness centrality values are bottlenecks. In addition, proteins that possess both features are assigned as hub-bottleneck agents. These elements are prominent for the network integrity, in which any small changes in these proteins may result in irregularity of protein systematic functions, and consequently a possible abnormal biological response in an organism. The actions between top 20 hub proteins are also determined as a nested network by the use of Clue Pedia. It is a Cytoscape Plug-in up to date. The action types that were determined in this study are activation, expression, and inhibition. The cutoff kappa score for this analysis was set to 0.5. Moreover, a cerebral view of the selected proteins can be helpful to understand the related cell components. For this purpose, 1. Extracellular, 2.Plasma membrane, 3.Intra Cellular, 4.Nuclear Membrane, 5.Nucleus, 6.Transcription Factor Complex were defined (23). Furthermore, for functional enrichment, ClueGO (23) analyzed the associated pathways for the top 20 hub proteins. The pathway sources obtained from the data were integrated by KEGG, WIKIPATHWAYS, and REACTOME databases. In a way that, a cut off of 0.5 was set for kappa score and terms including at least 3 genes were retrieved. The similar terms were grouped as clusters of pathways with p≤ 0.05. Each group was labeled by the name of the significant associated term.

## Results

**Figure 1:**
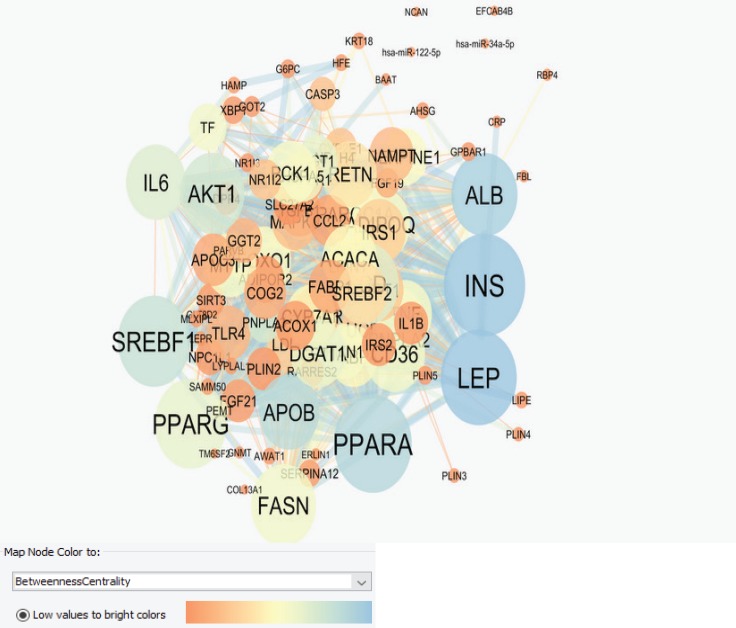
**.** Illustration of the protein-protein interaction network of Nash Disease with 100 nodes and 939 edges. This network has four isolated nodes. The color changes indicate BC Values as highlighted in the box below the figure. The node size corresponds to the degree value so bigger size corresponds to bigger degree

**Figure 2:**
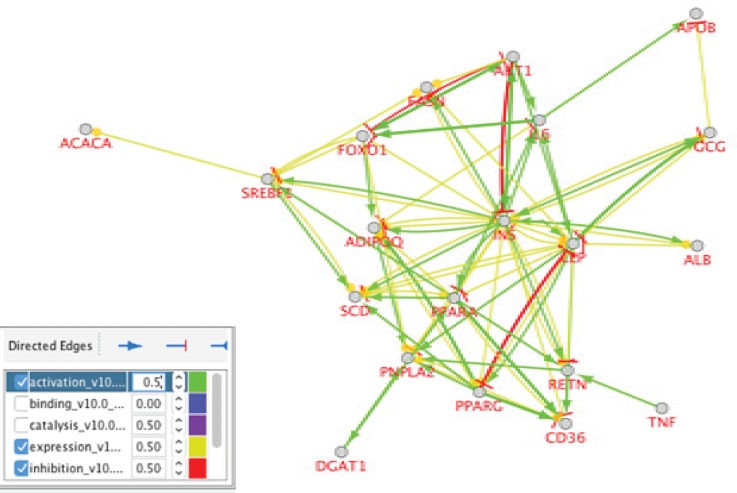
Activation, expression, and inhibition pattern of 20 first hubs of the main network in a nested network. The color and correspond description were shown in the box below the figure

**Figure 3.**
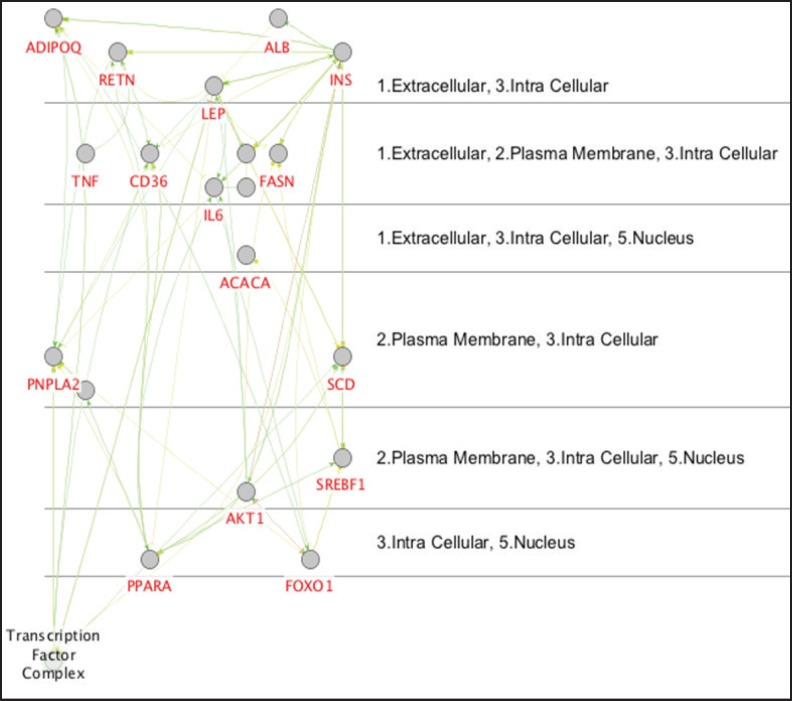
Cerebral view of the 20 first hub proteins of the main network illustrated in this figure. The cell components of these elements were assigned. The numbers were corresponding to the cell components

**Figure 4:**
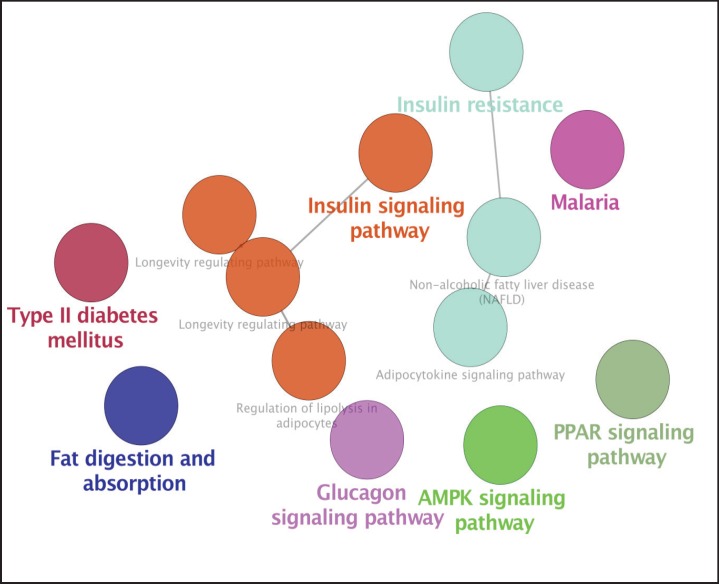
Pathways of the 20 first hubs of the network were illustrated in this figure. The kappa score was set to 0.5. The data integrated from KEGG, WIKIPATHWAYS, and REACTOME Databases

Cytoscape Software performed protein-protein interaction network analysis of Nash Disease. The dataset was derived from String Database, disease query (see figure1). The key proteins including the hub proteins and bottlenecks were determined. The top ten hub proteins were tabulated in table 1. For more resolution, the activation, expression, and inhibition pattern of 20 first hubs of the main network in a nested network presented in figure 2. Since the cell component is an important characteristic of the local place of a protein, the schema of cell component for the 20 first hub proteins of the main network showed in the figure 3. The involved pathways of the 20 first hubs of the network were analyzed and illustrated in figure 4.

## Discussion

As indicated earlier, fatty liver disease is a widespread liver condition around the world (24). There are many reported documents about NASH especially focused on its molecular aspects (16, 25, 26). Since the obtained data should be evaluated for applying in the field, PPI network analysis is one of the excellent methods for ranking and categorizing of the involved proteins in a disease (27). More resolution is available by pathway analysis of the distinguished proteins (28). The network was constructed by 100 related proteins mentioned in fig1. However, the main network contains 96 nodes and 4 nodes were excluded because they had no connection to the main network. There were 939 links per 96 nodes in the network. So the mean value of the edges per one node is about 10. The inhomogeneous distribution of the edges indicates the scale free characteristic of PPI network. The top ten proteins with highest degree were identified and tabulated in table 1. Degree as one of the centrality indices, corresponds to the links of a node to the other members of the network (28). A node with highest degree (hub protein) plays a crucial role in a network so its expression changes effect grossly on the function of the whole network (27). There are many documents that confirm relationship between the represented hub proteins in table one and NASH (29-33). Yet, their significant roles in this disease need more resolution. The bottleneck nodes are the proteins that effectively play role in the integrity of the network (27). As it is shown in the table 1, five hub proteins are bottleneck nodes.

**Table 1:** The top ten key proteins of the Nash PPI Network were introduced. The asterisked nodes are the hub-bottleneck proteins

**Protein Name**	**Disease Score**	**Degree**	**BC**
*Insulin	2.91	64	0.07
*Peroxisome proliferator-activated receptor alpha	2.43	60	0.06
*Leptin	2.62	58	0.08
*Sterol regulatory element binding transcription factor 1	3.10	54	0.05
Peroxisome proliferator-activated receptor gamma	2.43	53	0.03
*Albumin	1.68	47	0.07
Fatty acid synthase	1.79	46	0.02
Stearoyl-CoA desaturase (delta-9-desaturase)	2.42	46	0.03
V-akt murine thymoma viral oncogene homolog 1	1.70	45	0.04
CD36 molecule (thrombospondin receptor)	1.46	41	0.02

The disease score of these proteins also show a considerable relationship between them and fatty liver disease. Therefore, it is possible that an informative panel including 5 hub- bottleneck proteins to be introduced for NASH. Furthermore, a sub- network including the first 20 hub proteins is constructed (see figure 2). There are several important points about this sub- network:

A) Insulin is a key protein and linked to the all hub- bottleneck proteins.

B Insulin is activator for the mentioned proteins.

- Except a few links, all of the relationships between the nodes of the sub-network are activating effectors.
- Expression of the all hub-bottleneck proteins is affected by insulin expression changes.

Cell component analysis demonstrated that all of the crucial proteins including INS, PPARA, LEP, SREBF1, and ALB are intracellular proteins (fig3). However, INS, LEP, and Albumin are either extracellular protein. The presence of PPARA and SREBF1 in nucleus was also reported. It can be interpreted that mutual regulatory effects of these proteins and their presence in the various parts of the cell lead to involvement of many biochemical pathways. The enrichment analysis of the pathway (figure 4) introduced 8 highlighted involved pathways for the 20 first hubs of the network. These pathways are insulin signaling pathway, insulin resistance, glucagon signaling pathway, type II diabetes mellitus, AMPK signaling pathway, PPAR signaling pathway, and malaria. It seems that insulin plays a crucial role in pathology of fatty liver disease. Beside insulin involved pathways, glucagon signaling pathway also is a prominent pathway related to the disease. The role of insulin and glucagon in the glucose metabolism is highlighted in many documents (34, 35). These evidences and considering the roles of the other introduced pathways indicate that these key proteins are related closely to the fatty liver disease. The significant role of insulin in the analyzed network implies definition of a crucial role for insulin as like as its role in the diabetes. However, more investigations in the field is required. The findings lead to introduction of an informative biomarker panel including INS, PPARA, LEP, SREBF1, and ALB proteins related to the fatty liver disease. Pathway analysis showed significant role of insulin in development of disease and closed relationship between the highlighted biomarker panel and fatty liver disease.
